# Supplementary material for: Causal Relationship between Aging and Anorexia Nervosa: A White-Matter-Microstructure-Mediated Mendelian Randomization Analysis
Source: Biomedicines. 2024 Aug 16;12(8):1874. doi: 10.3390/biomedicines12081874 (PMC11351342; doi:10.3390/biomedicines12081874)
Supplement: Supplementary file 1 [file biomedicines-12-01874-s001.zip › Supplementary Figure/Figure S4/Singe SNP plot of MR analysis for TL to ubm-b-1987.pdf]

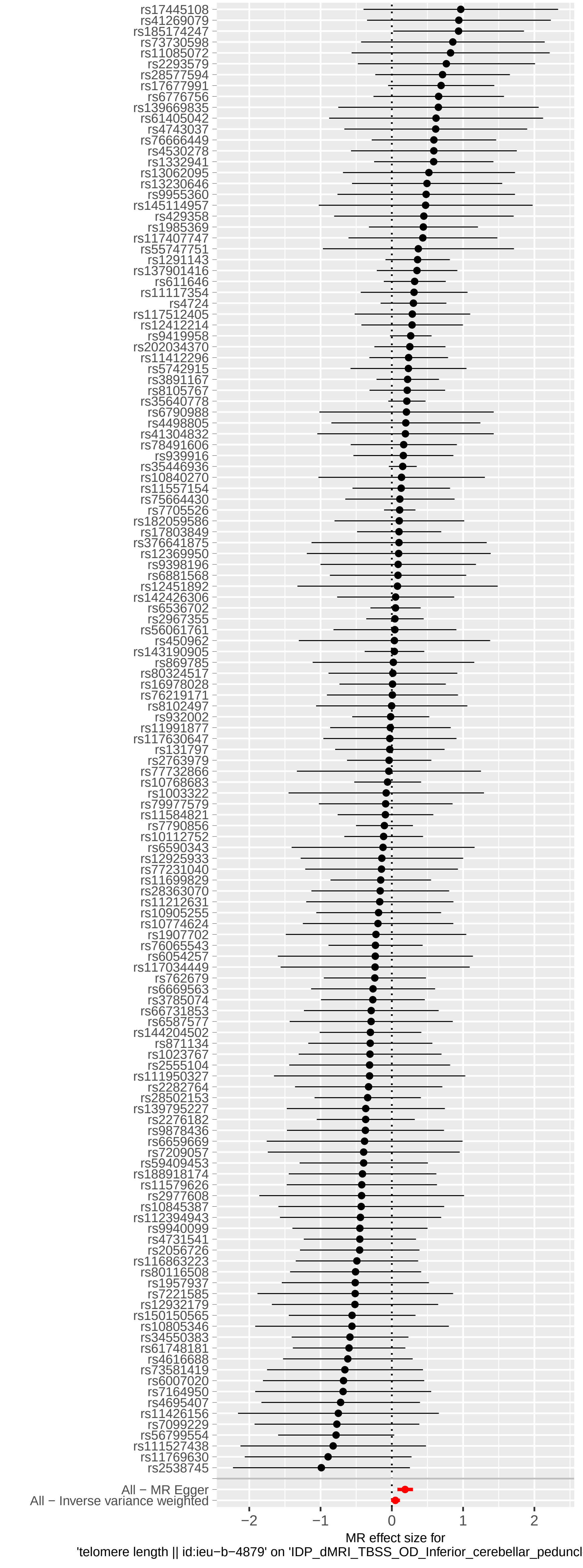
rs17445108
rs41269079
rs185174247
rs73730598
rs11085072
rs2293579
rs28577594
rs17677991
rs6776756
rs139669835
rs61405042
rs4743037
rs76666449
rs4530278
rs1332941
rs13062095
rs13230646
rs9955360
rs145114957
rs429358
rs1985369
rs117407747
rs55747751
rs1291143
rs137901416
rs611646
rs11117354
rs4724
rs117512405
rs12412214
rs9419958
rs202034370
rs11412296
rs5742915
rs3891167
rs8105767
rs35640778
rs6790988
rs4498805
rs41304832
rs78491606
rs939916
rs35446936
rs10840270
rs11557154
rs75664430
rs7705526
rs182059586
rs17803849
rs376641875
rs12369950
rs9398196
rs6881568
rs12451892
rs142426306
rs6536702
rs2967355
rs56061761
rs450962
rs143190905
rs869785
rs80324517
rs16978028
rs76219171
rs8102497
rs932002
rs11991877
rs117630647
rs131797
rs2763979
rs77732866
rs10768683
rs1003322
rs79977579
rs11584821
rs7790856
rs10112752
rs6590343
rs12925933
rs77231040
rs11699829
rs28363070
rs11212631
rs10905255
rs10774624
rs1907702
rs76065543
rs6054257
rs117034449
rs762679
rs6669563
rs3785074
rs66731853
rs6587577
rs144204502
rs871134
rs1023767
rs2555104
rs111950327
rs2282764
rs28502153
rs139795227
rs2276182
rs9878436
rs6659669
rs7209057
rs59409453
rs188918174
rs11579626
rs2977608
rs10845387
rs112394943
rs9940099
rs4731541
rs2056726
rs116863223
rs80116508
rs1957937
rs7221585
rs12932179
rs150150565
rs10805346
rs34550383
rs61748181
rs4616688
rs73581419
rs6007020
rs7164950
rs4695407
rs11426156
rs7099229
rs56799554
rs111527438
rs11769630
rs2538745
All − MR Egger
All − Inverse variance weighted
−2
−1
0
1
2
MR effect size for
'telomere length || id:ieu−b−4879' on 'IDP_dMRI_TBSS_OD_Inferior_cerebellar_peduncl
